# Supplementary material for: Controlled Synthesis of Au Nanocrystals-Metal Selenide Hybrid Nanostructures toward Plasmon-Enhanced Photoelectrochemical Energy Conversion
Source: Nanomaterials (Basel). 2020 Mar 20;10(3):564. doi: 10.3390/nano10030564 (PMC7153708; doi:10.3390/nano10030564)
Supplement: Supplementary file 1 [file nanomaterials-10-00564-s001.pdf]

nanomaterials

Supporting Information

Controlled Synthesis of (Au Nanocrystals)-(Metal Selenide) Hybrid Nanostructures toward Plasmon-Enhanced Photoelectrochemical Energy Conversion

Ling Tang [1], Shan Liang [1,2,*], Jianbo Li [3,4,*], Dou Zhang [5], Wenbo Chen [1], Zhongjian Yang [5], Si Xiao [5], and Ququan Wang [6]

1 Department of Physics, Hunan Normal University, Changsha 410081, P.R. China; 1924411950@qq.com (L.T.); 1251546091@qq.com (W.C.)
2 Key Laboratory for Matter Microstructure and Function of Hunan Province, Hunan Normal University, Changsha 410081, China
3 Institute of Mathematics and Physics, Central South University of Forestry and Technology, Changsha 410004, China
4 School of materials science and energy engineering, Foshan University, Foshan, Guangdong Province, 528000, China
5 Hunan Key Laboratory of Super Microstructure and Ultrafast Process, School of Physics and Electronics, Central South University, Changsha 410083, China; 1309830571@qq.com (D.Z.); zjyang@csu.edu.cn (Z.Y.); sixiao@csu.edu.cn (S.X.)
6 Department of Physics, Key Laboratory of Artificial Micro-and Nano-Structures of the Ministry of Education, Wuhan University Wuhan 430072, P.R. China; qqwang@whu.edu.cn (Q.W.)
* Correspondence: liangshan@hunnu.edu.cn (S.L.); jbli_opt@csuft.edu.cn (J.L.)

Keywords: gold-metal selenide; hollow hybrid nanostructure; surface plasmon resonance; morphology manipulation; photoelectrochemical response

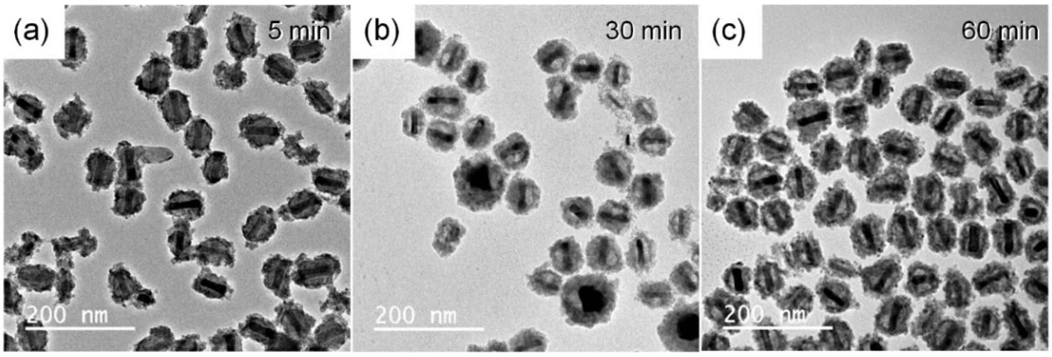

Figure S1. Low magnification TEM images of the products obtained at different reaction time for synthesis of yolk-shell Au−PbSe NRs.

Synthesis of Au-PbSe Hybrids with Different Thicknesses of Se Shells

Figure S2 demonstrates that the sizes of hollow nanocavity and semiconductor nanoshell for Au-PbSe yolk-shell hybrid NRs could be tuned by the sizes of Se shells. When the added amount of Se source was 5 μL (or 10 μL) of 0.1 M, the corresponding average thickness of Se shell was about 4.2 nm (or 11.3 nm). Subsequently, as 20μL of 0.1 M Pb^{2+} precursor was added, Au-PbSe yolk-shell hybrid NRs with different shell thicknesses and hollow sizes were achieved. As shown in Figure S2, the obtained Au-PbSe NRs kept yolk-shell nanostructure, the average thicknesses of PbSe shells were 4.3 nm and 8.5 nm, and the corresponding hollow sizes were about 0.4 nm and 4.8 nm respectively.

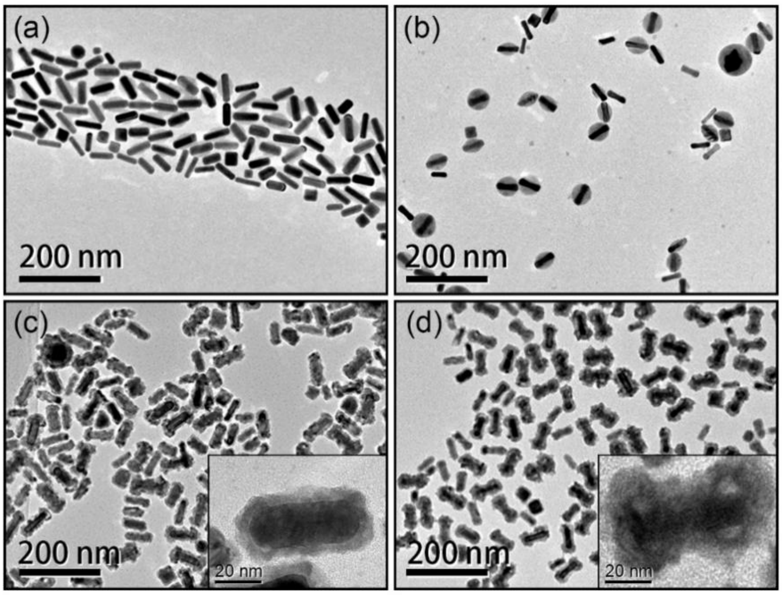

Figure S2. The average shell thicknesses of Au@Se NRs were respectively 4.2 nm and 11.3 nm. When 20 μL of 0.1 M lead acetate solution was added, the obtained Au-PbSe NRs kept yolk-shell nanostructure, the average thicknesses of PbSe shells were 4.3 nm and 8.5 nm, and the corresponding nanovoid sizes were about 0.4 nm and 4.8 nm respectively.

Synthesis of Au-CdSe, Au-Bi_2Se_3 and Au-CuSe Hybrids

In the same method, Au-CdSe, Au-Bi_2Se_3 and Au-CuSe Hybrid NRs were also achieved, by using the corresponding Gly[M^{n+}] (M^{n+} = Cd^{2+}, Bi^{3+} and Cu^{2+}) precursors instead of Gly[Pb^{2+}] complexes, respectively. The details are as follows: (i) in order to prepare Au-CdSe hybrid NRs, Gly[Cd^{2+}] precursor was prepared by mixing 0.002 mL of 0.1 M $Cd(C_2H_3O_2)_2{\cdot}3H_2O$ and 1.0 mL of 0.2 M Gly in a 10 mL round-bottom test tube, followed by addition of 1 mL of the as-prepared Au@Se NRs seed. The *pH* of the reaction solution was regulated to 8.5 with 2 M NaOH solution. Subsequently, 0.02 mL of 0.1 M AA was added and kept at 80 °C under vigorously stirring for 90 min. (ii) to prepared Au-Bi_2Se_3 hybrid NRs, Gly[Bi^{3+}] precursor was prepared by mixing 0.013 mL of 0.01 M $Bi(NO_3)_3{\cdot}5H_2O$ and 1.0 mL of 0.2 M Gly in a 10 mL round-bottom test tube, followed by the addition of 1 mL of the as-prepared Au@Se NRs seed. Subsequently, 0.02 mL of 0.1 M AA was added and kept at 60 °C under vigorously stirring for 300 min. (iii) to prepare Au-CuSe hybrid NRs, Gly[Cu^{2+}] precursor was prepared by mixing 0.002 mL of 0.1 M $CuCl_2$ and 1.0 mL of 0.2 M Gly in a 10 mL round-bottom test tube, followed by the addition of 1 mL of the as-prepared Au@Se NRs seed. The *pH* of the reaction solution was regulated to 9 with 2 M NaOH solution. Subsequently, 0.02 mL of 0.1 M AA was added and kept at 80 °C under vigorously stirring for 90 min. The final products were centrifuged at 8000 rpm for 9 min, washed with water for two times, and dispersed in water solution. For comparison, PbSe NCs was obtained by the same method except using the corresponding CTAB (0.1 M) aqueous solution instead of Au NRs.

Figure S3 displays the morphology and detailed crystallographic structures of the corresponding hybrid NRs. As shown in Figures S3a-c, according to the XRD pattern analysis shown in Figure S3(h), the diffraction peaks from CdSe, Bi_2Se_3 and CuSe can be, respectively, well identified according to JCPDS cards (nos. 08-0459, 33-0214 and 20-1020), which indicates that the crystalline phases of the semiconductor shells are all hexagonal forms. EDS analysis in Figure S3(g) verifies the element composition of Au-CdSe and Au-Bi_2Se_3 hybrid NRs. The corresponding HRTEM images at the ends of single individual hybrid nanoparticles exhibit three distinct sets of lattice fringes, which can be, respectively, indexed to the {100} reflection of hexagonal CdSe, {110} reflection of hexagonal Bi_2Se_3, and {101} reflection of hexagonal CuSe.

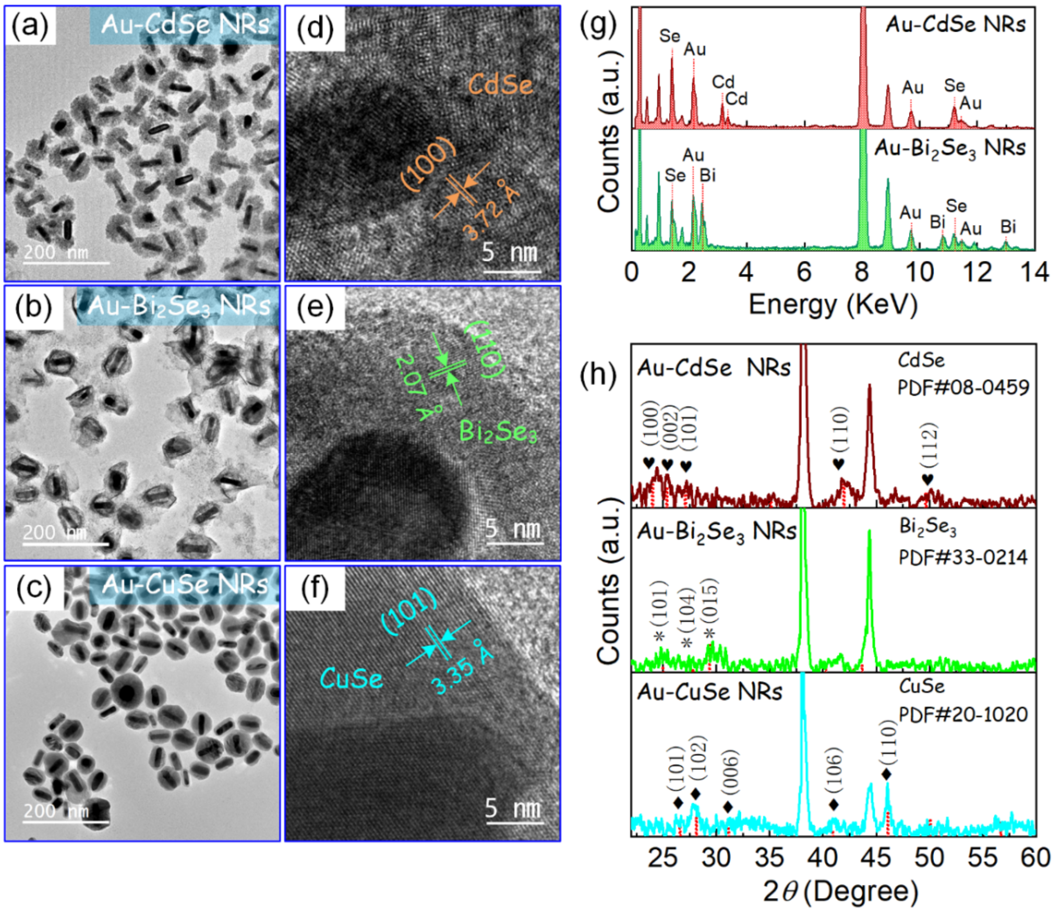

Figure S3. TEM images of (**a**) Au-CdSe NRs, (**b**) Au-Bi_2Se_3 NRs and (**c**) Au-CuSe NRs. (**d-f**) corresponding HRTEM images at the ends of hybrid NR, (**g**) EDS spectra of Au-CdSe NRs and Au-Bi_2Se_3 NRs, (**h**) XRD patterns of Au-CdSe NRs, Au-Bi_2Se_3 NRs and Au- CuSe NRs. The standard patterns of hexagonal-phase CdSe (PDF#08-0459), Bi_2Se_3 (PDF#33-0214) and CuSe (PDF#20-1020) are also displayed.

Synthesis of Au-PbSe Hybrids Nanocubes and Triangular Nanoprisms

The initial Au nanocubes and triangular nanoprisms were synthesized by seed-mediated methods reported in the previous works [1,2], 1 mL Au nanoparticles obtained was centrifuged at 9500 rpm for 9 min and redispersed in 0.2 M CTAB solution with the same volume for further use. Au@Se nanostructures prepared by a one-pot method. In brief, the above Au nanoparticles and 0.05 mL of 0.1 M AA were firstly mixed in a 10 mL round-bottom test tube. After stirring for 3 min, 0.02 mL of 0.1 M SeO_2 was added. The mixed solution was then incubated at 38 °C with evenly stirring for 5 h for synthesizing Au@Se seeds. The obtained samples were then centrifuged at 9500 rpm for 9 min, and redispersed in 1 mL of 0.2 M CTAB solution. Subsequently, Gly[Pb^{2+}] which acts as the Pb^{2+} precursor was prepared by 0.02 mL of 0.1 M $Pb(C_2H_3O_2)_2{\cdot}3H_2O$ and 1.0 mL of 0.2 M glycine in a 10 mL round-bottom test tube, followed by addition of 1 mL of the as-prepared Au@Se seed. The *pH* of the reaction solution was regulated to 9.8 with 2 M NaOH solution. Finally, 0.02 mL of 0.1 M AA was added and kept at 80 °C under vigorously stirring for 90 min. The obtained final products were then centrifuged at 8000 rpm for 9 min, and redispersed in water solution. Corresponding TEM images of the obtained Au-PbSe hybrid nanostructures were exhibited in Figure S4.

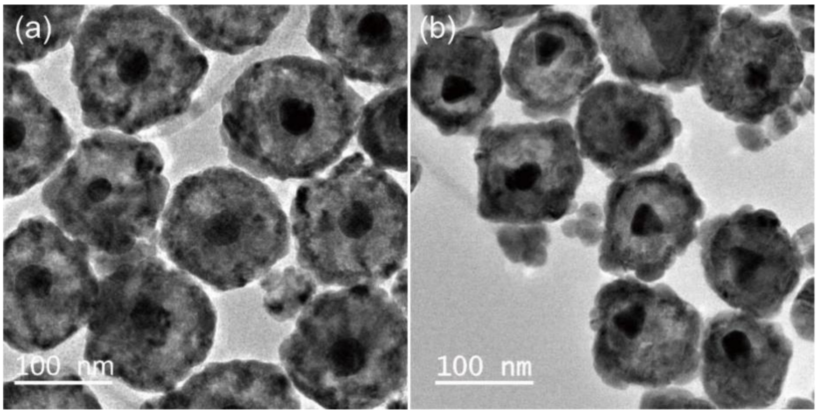

Figure S4. TEM images of Au-PbSe yolk-shell (**a**) nanocubes NCs and (**b**) triangular nanoprisms prepared by the same method.

FDTD Simulations

The simulations were carried out by using finite-difference time domain (FDTD) method with the software FDTD Solutions 8.6. The used refractive indexes of the semiconductor shells and surrounding matrix are 4.9 and 1.33, respectively. The dielectric constants of gold are taken from Refs [3]. The total-field scattered-field (TFSF) plane wave is used as the excitation plane wave source. Perfectly matched layer (PML) boundary conditions were used to simulate an individual structure placed in an infinite space.

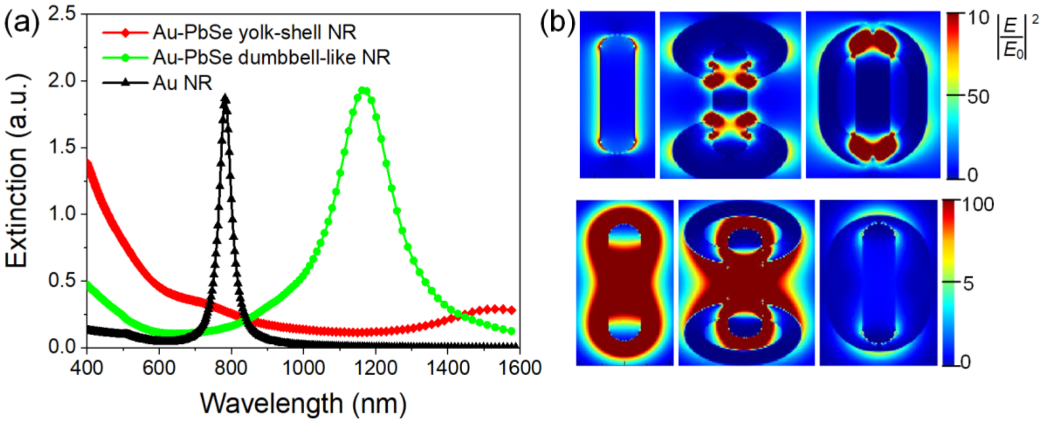

Figure S5. (**a**) Simulated extinction spectra of Au NR, Au-PbSe dumbbell-like and yolk-shell NRs with different symmetric hollow shells; (**b**) Local field distributions for these three nanostructures at their T-SPR and L-SPR peaks.

Photoactivity of (Au-PbSe Hybrid NRs)@TiO_2 Photoanodes

All sensitizers had the same mass of corresponding components as described in the table S1. PbSe NCs with hollow nanostructure were prepared by the same method, except using the CTAB (0.1 M) aqueous solution instead of Au NRs. Their TEM images are shown in Figure S6, the average shell thickness of Au-PbSe yolk-shell NRs is about 4.3 nm, and the size of semiconductor counterpart is about 27.5 nm for Au-PbSe dumbbell NRs. The typical scanning electron microscope (SEM) image of (Au-PbSe yolk-shell NRs)@TiO_2 electrode was shown in Figure S8. The thickness of TiO_2 NWs film is about 338.8 nm. Au-PbSe NRs are successfully loaded on the surface of TiO_2 NWs. Photocurrent responses of four parallel photoanodes are assessed in a three-electrode cell at 0.1 V versus Ag/AgCl, and sulfite solution was employed as sacrificial hole scavenger.

Table S1. The addition amount of reaction precursors are shown in the table respectively for synthesizing sensitizer of four parallel photoanodes.

	as-prepared Au NRs	SeO_2 (0.1 M)	$Pb(C_2H_3O_2)_2 \cdot 3H_2O$
TiO_2 NWs substrate			
(PbSe NCs)@TiO_2	1 mL	20 μL	5 μL
(Au-PbSe dumbbell NRs)@TiO_2	1 mL	20 μL	5 μL
(Au-PbSe yolk-shell NRs)@TiO_2	1 mL	5 μL	20 μL

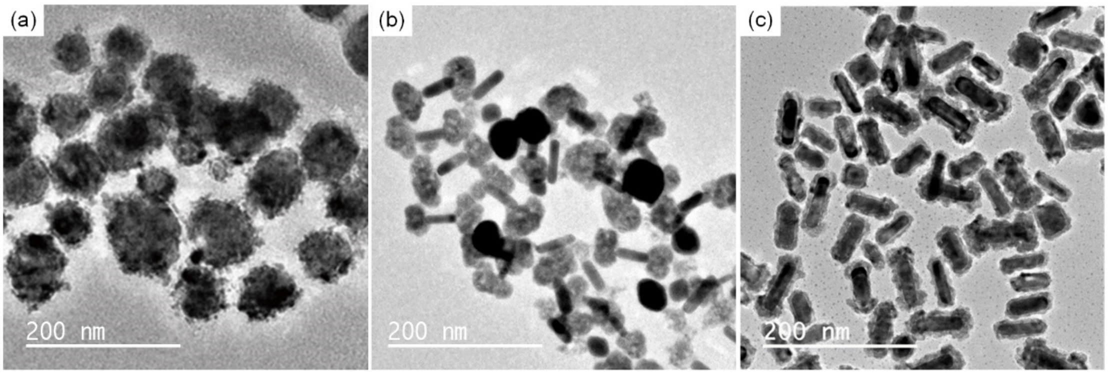

Figure S6. Corresponding TEM images of three sensitizers (**a**) hollow PbSe NCs; (**b**) Au-PbSe dumbbell-like and (**c**) yolk-shell NRs.

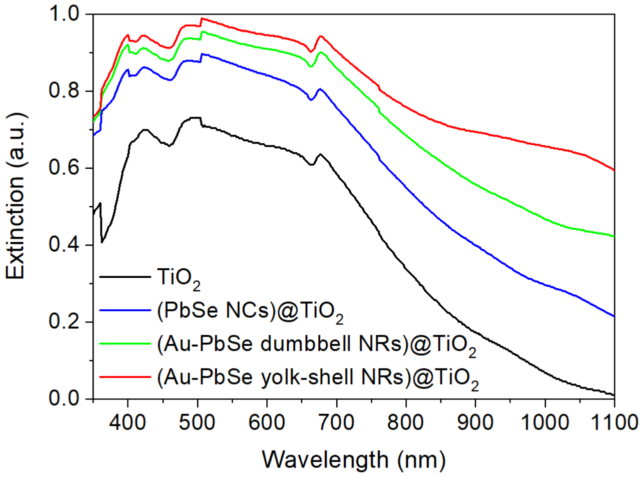

Figure S7. Absorption spectra of pristine TiO_2, (PbSe NCs)@TiO_2, (Au-PbSe dumbbell NRs)@TiO_2 and (Au-PbSe yolk-shell NRs)@ TiO_2.

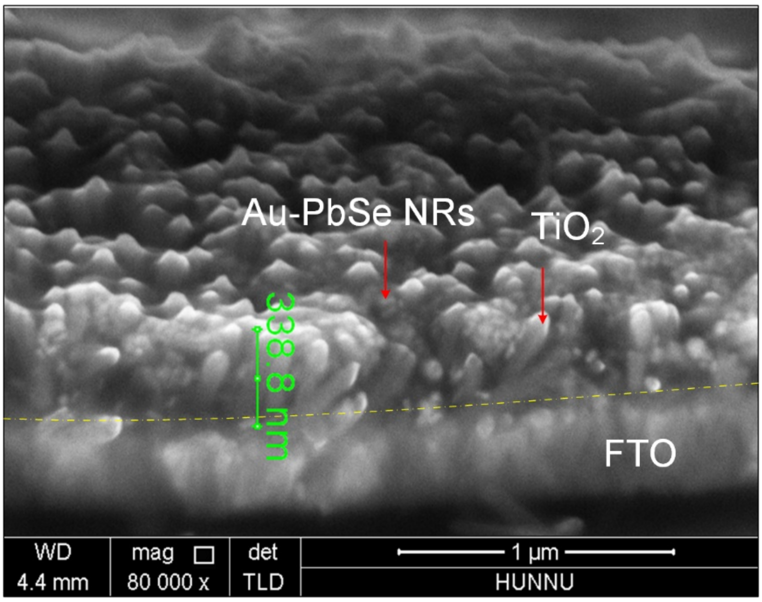

Figure S8. SEM image of TiO_2 NWs photoanodes sensitized with Au-PbSe yolk-shell NRs.

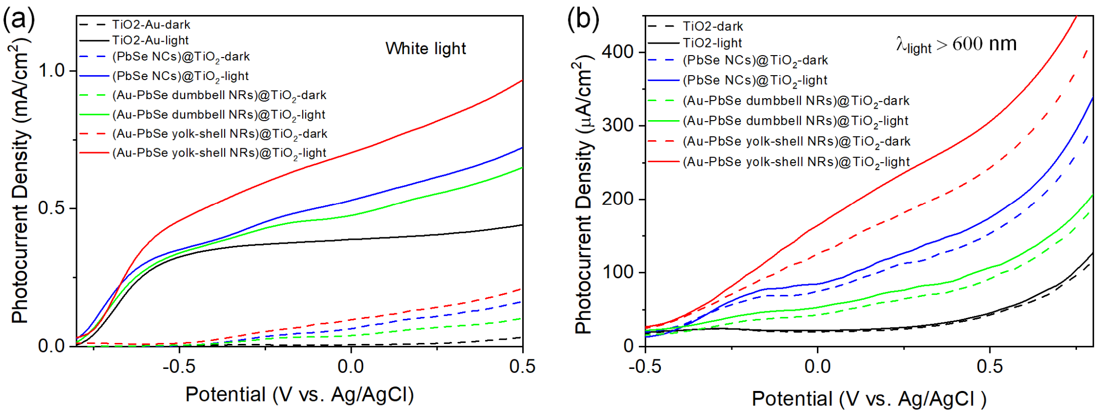

Figure S9. Photocurrent density-applied potential characteristics under illuminations of white light **(a)** and **(b)** the light at $\lambda > 600$ nm.

Incident photon to current conversion efficiency

The incident-photon-to-current-conversion efficiency (IPCE) of (PbSe NCs)@TiO_2 and (Au-PbSe yolk-shell NRs)@TiO_2 was measured by using a 150 W Xenon lamp equipped with a monochromator to get the desired incident wavelength (600-1000 nm). The working electrode area is 1 cm^2. Na_2SO_3 (0.25 M) and Na_2S (0.35 M) aqueous solution ($pH \approx 12$), Ag/AgCl, and Pt were employed as electrolyte, reference electrode, and counter electrode, respectively. The measurement parameters and results of (Au-PbSe yolk-shell NRs)@TiO_2 with incident light wavelength of 600 nm are given. The average intensity of irradiation was determined to be 0.607 $mW{\cdot}cm^{-2}$ and the photocurrent density was $5.62{\times}10^{-8}$ cm^2. IPCE is defined as the ratio between the number of electrons generated by internal and external circuits per unit time N_e and the number of photons of monochromatic light incident per unit time N_p, and its calculation formula is as follows:

$$\mathrm{IPCE}[\%] = \frac{Ne}{Np} \times 100, \text{and } Ne = \frac{iAt}{e}, Np = \frac{P_{in}At}{hc/\lambda}$$

$$\mathrm{IPCE}[\%] = \frac{1240i}{\lambda P_{in}} \times 100$$

Where, i is the photogenerated current density, λ is the wavelength of incident monochromatic light, A is the surface area of the photoelectric material, t is the irradiation time, e is the electron charge, c is the speed of light in vacuum, h is the Planck constant, and P_{in} is the irradiance of the surface of the photoelectric material. When λ = 600 nm：

$$\mathrm{IPC}E[\%] = \frac{1240i}{\lambda P_{in}} \times 100 = \frac{1240 \times 5.62 \times 10^{-2}}{600 \times 6.07} \times 100 = 1.91\%$$

The calculated IPCE at 600 nm was 1.91%.

1. Scarabelli, L.; Coronado, P. M.; Giner, C. J. J.; Langer, J.; Liz, M. L. M. Monodisperse gold nanotriangles: Size control, large-scale self-assembly, and performance in surface-enhanced raman scattering. *ACS nano* **2014**, *8*, 5833-5842.
2. Liu, X. L.; Liang, S.; Nan, F.; Yang, Z. J.; Yu, X. F.; Zhou, L.; Hao, Z. H.; Wang, Q. Q. Solution-dispersible Au nanocube dimers with greatly enhanced two-photon luminescence and SERS. *Nanoscale* **2013**, *5*, 5368-5374.
3. Johnson, P. B.; Christy R. W. Optical constants of the noble metals. *Phys. Rev. B.* **1972**, *15*, 4370.
